# Elevated Rates of Ventilator-Associated Pneumonia and COVID-19 Associated Pulmonary Aspergillosis in Critically Ill Patients with SARS-CoV2 Infection in the Second Wave: A Retrospective Chart Review

**DOI:** 10.3390/antibiotics11050632

**Published:** 2022-05-07

**Authors:** Sean Boyd, Kai Sheng Loh, Jessie Lynch, Dhari Alrashed, Saad Muzzammil, Hannah Marsh, Mustafa Masoud, Salman Bin Ihsan, Ignacio Martin-Loeches

**Affiliations:** 1 Multidisciplinary Intensive Care Research Organization (MICRO), St James’s Hospital, D08 NHY1 Dublin, Ireland; lohk@tcd.ie (K.S.L.); jessielynch993@gmail.com (J.L.); dharialrashed@rcsi.com (D.A.); drsaadsheikh@gmail.com (S.M.); hannahmarsh@rcsi.ie (H.M.); mustesharef@gmail.com (M.M.); binihsas@tcd.ie (S.B.I.); drmartinloeches@gmail.com (I.M.-L.); 2 Trinity College Dublin, University of Dublin, DN02 PN40 Dublin, Ireland; 3 Pulmonary Intensive Care Unit, Respiratory Institute, Hospital Clinic of Barcelona, IDIBAPS, University of Barcelona, CIBERes, 08036 Barcelona, Spain

**Keywords:** ICU, COVID-19, influenza, community-acquired pneumonia, VAP, IPA, CAPA, *Aspergillus*

## Abstract

Due to multiple risk factors, the rate of ventilator-associated pneumonia in critically ill COVID-19 patients has been reported in a range of 7.6% to 86%. The rate of invasive pulmonary aspergillosis in this cohort has been reported at 4% to 30%. We undertook a retrospective chart review of 276 patients who were admitted to intensive care in a large university hospital. The period studied included patients from 23 February 2014 to 12 May 2021. Four groups were collected: COVID-19 Wave 1, COVID-19 Wave 2, influenza, and community-acquired pneumonia. Clinical characteristics, outcomes, and microbiological cultures were recorded. The incidence of ventilator-associated pneumonia in COVID-19 Wave 1, COVID-19 Wave 2, influenza, and community-acquired pneumonia was 5.45%, 27.40%, 16.67%, and 3.41%, respectively (*p* < 0.001). The rate of invasive pulmonary aspergillosis was 0%, 9.59%, 13.33%, and 6.82%, respectively (*p* < 0.001). A significantly elevated rate of ventilator-associated pneumonia and invasive pulmonary aspergillosis was noted in the second wave of COVID-19 when compared to the first. This was accompanied by an increase in the mortality rate. Increased steroid use was an independent risk factor for ventilator-associated pneumonia and invasive pulmonary aspergillosis across all four groups. Despite an increased understanding of this disease, no clinical trials have shown any promising therapeutic options at present.

## 1. Background

Since the beginning of the COVID-19 pandemic, intensive care units worldwide have been put to the test. Different issues relating to the disease have posed new challenges to clinicians. These include higher ventilator-associated pneumonia rates [1,2,3,4,5] and invasive pulmonary aspergillosis rates [6,7,8]. As these patients are more likely to require intensive care along with prolonged mechanical ventilation, they are at an elevated risk of developing ventilator-associated pneumonia [9,10]. The use of immunomodulating agents such as corticosteroids, IL-6 inhibitors, and Janus kinase inhibitors may all increase this risk [11]. Yet, some evidence suggests improved outcomes with steroids [12,13]. Critically ill COVID-19 patients have been shown to have more severe parenchymal damage and poor lung compliance when compared to non-COVID-19 patients [14]. It is thought that COVID-19 causes dysregulation of the immune system and lymphoid function, resulting in hyperinflammation [15,16,17,18]. Studies have shown changes in the lung microbiome of COVID-19 patients, along with a decrease in the antibacterial function of their immune system [19,20]. Pulmonary vascular endothelial inflammation and thrombosis may occur at higher rates in this cohort of patients, yielding favourable conditions to support bacterial growth [21,22,23,24,25]. Invasive pulmonary aspergillosis has been associated with conditions such as COPD, ARDS, cirrhosis, acute hepatitis, and immunosuppression [26]. Patients requiring invasive mechanical ventilation have been deemed high risk. It has been shown that in both critically ill influenza and COVID-19 patients, invasive pulmonary aspergillosis has been associated with increased ICU length of stay, length of mechanical ventilation, and ICU mortality [26].

At the beginning of the pandemic, adequate staffing and sufficient personal protective equipment may not have been available for intensive care units. This could have increased the rate of cross-contamination. This may have subsequently increased the rates of ventilator-associated pneumonia [8,9]. The reported rate of ventilator-associated pneumonia in COVID-19 patients is between 7.6% and 86% [9]. The reported rate of COVID-19 associated pulmonary aspergillosis is reported as 4% to 30% [8,27]. The mortality rate of COVID-19 patients who develop ventilator-associated pneumonia is estimated at 42.7% [28]. Although studies suggest an increased mortality rate, it has been difficult to quantify the exact mortality rate of patients who develop COVID-19 associated pulmonary aspergillosis [8]. These infections pose a serious issue for this cohort of patients.

## 2. Methods

We performed a retrospective chart review of 276 patients that had been admitted to the intensive care unit in a large university hospital in Ireland. This included the following four groups:

1. The first wave of COVID-19: This group was admitted from 15 March 2020 to 24 May 2020 (*n* = 55).
2. The second wave of COVID-19: This group was admitted from 31 August 2020 to 13 February 2021 (*n* = 73).
3. All influenza patients admitted from 23 February 2014 to 03 February 2020 (*n* = 60).
4. All community-acquired pneumonia patients admitted from 11 January 2019 to 12 May 2021 (*n* = 88).

All patients included in both waves of COVID-19 and the influenza group had a polymerase chain reaction confirmed diagnosis of SARS-CoV2 or influenza, respectively. The community-acquired pneumonia group was identified based on clinical diagnosis. In order to study both the influenza and community-acquired pneumonia groups, the number of patients needed to be similar to that of the COVID-19 groups. As a result, patients from previous years were included in the study.

The study aimed to quantify and compare ventilator-associated pneumonia rates and invasive pulmonary aspergillosis rates between the four groups.

Ventilator-associated pneumonia was diagnosed based on local guidelines and as per physician discretion. Fever, cough, hypoxaemia, deterioration, new chest X-ray findings, and positive microbiological cultures were all included in the diagnostic criteria. An element of subjectivity does exist, and there can be variation between clinicians. All patients diagnosed with ventilator-associated pneumonia had been intubated at least 48 h prior. Only the first episode of ventilator-associated pneumonia was included. Invasive pulmonary aspergillosis was diagnosed based on a positive culture of *Aspergillus* on bronchoalveolar lavage, endotracheal aspirate, or sputum sample. Radiological criteria were not used.

The following criteria were used to determine immunosuppression: solid tumour with chemotherapy in the last 3 months, progressive metastatic disease, haematological malignancies, solid organ transplantation, HIV infection (with or without AIDS), corticosteroids (>3 months at any dosage or ≥1 mg/kg prednisone equivalent per day for >7 days), and/or immunosuppressive drugs.

SAPSII (simplified acute physiology score) and SOFA (sequential organ failure assessment) scores were recorded for all groups. These scores are utilised to estimate mortality.

We used SPSS (version 20) for data analysis. All *p*-values were two-tailed. We considered differences as significant if *p* was less than 0.05. We reported categorical variables as numbers and frequencies (%), normally distributed continuous variables as means (standard deviation [SD]), and skewed continuous variables as medians (interquartile range [IQR]). We performed both χ^2^ tests or Fisher’s exact tests to compare qualitative variables and Student’s *t*-tests and ANOVAs or Mann–Whitney U and non-parametric Kruskal–Wallis tests to compare normally distributed or skewed continuous variables, whenever appropriate. In order to determine the independent risk factors for ventilator-associated pneumonia and invasive pulmonary aspergillosis development, a multivariate analysis was performed, including variables with *p* < 0.1 and adjusted by the time under invasive mechanical ventilation (by day) using a backward method. Wave 1 was considered the reference value.

This study has been deemed minimal-risk research using data collected for routine clinical practice, and therefore, the requirement for informed consent has been waived. Reference: REC: 2020-05 List 17.

## 3. Results

A comparison of the clinical characteristics of the four groups is displayed in Table 1. There was a male predominance throughout all groups (*p* = 0.381). All four groups had similar mean age, co-morbidities, and BMI, for the most part. The influenza group had a significantly elevated rate of ischaemic heart disease when compared to the other groups (*p* < 0.001). Patients in the second wave of COVID-19 had a significantly increased rate of hypertension in comparison to the other groups (*p* = 0.012). The community-acquired pneumonia group and the influenza group had significantly elevated rates of chronic obstructive pulmonary disease compared to the other groups (*p* = 0.003).

The mean SAPSII scores [SD] for COVID-19 Wave 1, COVID-19 Wave 2, influenza and community-acquired pneumonia were 49.75 [18.63], 41.63 [17.63], 55.73 [17.26], 48.92 [18.83] respectively (*p* < 0.001). The mean SOFA scores [SD] for COVID-19 Wave 1, COVID-19 wave 2, influenza and community-acquired pneumonia were 9.18 [4.32], 8.94 [4.77], 11.13 [4.49], 9.76 [4.15], respectively (*p* = 0.029). It is evident that there was a significant increase in both SAPSII and SOFA scores in the influenza group when compared to the other groups.

The use of steroids was significantly elevated in the second wave of COVID-19 when compared to the first wave of COVID-19 (*p* < 0.001). Antifungal therapy was significantly elevated in the second wave of COVID-19 in comparison to the first wave of COVID-19 (*p* < 0.001). CPIS (clinical pulmonary infection scores) [SD] were as follows: 2.00 [0], 4.46 [3.15], 1.52 [2.62], 6.67 [4.16], respectively (*p* <0.001). The first wave of COVID-19 and the influenza groups were significantly lower than the second wave of COVID-19 and the community-acquired groups.

In terms of outcomes (Table 2.), there was a significant increase in ICU length of stay (*p* = 0.010), length of mechanical ventilation (*p* = 0.009), and ICU mortality (*p* = 0.047) in the second wave of COVID-19 when compared to the first.

The rates of ventilator-associated pneumonia (*p* < 0.001) and invasive pulmonary aspergillosis were significantly elevated in the second wave of COVID-19 (*p* < 0.001). This may have been related to an increase in steroid use seen in the second wave (*p* < 0.001) (Table 1.). A multivariate analysis was performed, and the independent risk factors associated with the development of ventilator-associated pneumonia (Hosmer-Lemeshow *p* = 0.54) were the use of steroids (OR 5.24, 95% CI 1.11–24.38) and the second wave (OR 7.82, 95% CI 7.88–34.68) during COVID-19. For the presence of invasive pulmonary aspergillosis (Hosmer–Lemeshow *p* = 0.47), only the use of corticosteroids (OR 11.11, 95% CI 1.38–9.27) was the independent risk factor when the multivariate analysis was performed (Supplementary Material Tables S1 and S2).

Causative agents cultured in the COVID-19 Wave 1, COVID-19 Wave 2, influenza, and community-acquired pneumonia groups are displayed in Table 3.

There was no evidence of any atypical bacterial growth in both waves of COVID-19 and the community-acquired pneumonia group. One patient in the second wave of COVID-19 with ventilator-associated pneumonia grew *Streptococcus* as the causative agent. The influenza group showed a rate of 5% co-infection with *Pneumococcus* on admission, but not as the cause of ventilator-associated pneumonia.

## 4. Discussion

In this study, we found a significant elevation in the rate of ventilator-associated pneumonia and invasive pulmonary aspergillosis in the second wave of COVID-19 when compared to the first wave. A higher ICU mortality rate accompanied this. The patients in the second wave of COVID-19 had a longer ICU length of stay and length of mechanical ventilation. There was a significant increase in steroid use, which was found to be an independent risk factor for the development of ventilator-associated pneumonia and invasive pulmonary aspergillosis across all four groups.

A recent meta-analysis by Ippolito et al. has reported ventilator-associated pneumonia rates in critically ill COVID-19 patients at approximately 45.4%, with a range of 7.6% to 86%. This wide range could result from different clinical settings and staffing levels. The individual studies included in the meta-analysis may have dealt with different patient populations and different severities of illness. An important factor to consider is the variations in diagnostic criteria [9]. The rate of ventilator-associated pneumonia in our intensive care unit in the first wave of COVID-19 was 5.45%, and 27.40% in the second wave (*p* < 0.001). This is on the lower end of the range quoted by Ippolito et al. It is possible that the overall lower rates of ventilator-associated pneumonia in COVID-19 patients in our intensive care unit are a result of the same factors that caused such a wide variation in the meta-analysis. Yet, the rate of ventilator-associated pneumonia was still significantly elevated in the second wave compared to the first. Fumagalli et al. described the rate of invasive pulmonary aspergillosis in COVID-19 patients to be 4% to 30% [8]. The rates of invasive pulmonary aspergillosis in our study were 0% and 9.59% in the first and second waves of COVID-19, respectively (*p* < 0.001). This is in keeping with the findings reported by Fumagalli et al. A significant difference between the first and second waves of COVID-19 was seen in our intensive care unit.

Fumagalli et al. reported the mean length of mechanical ventilation in COVID-19 patients that developed ventilator-associated pneumonia as twelve to thirty days [8]. Our study’s median length of mechanical ventilation in days was 8.00 in the first wave of COVID-19, and 11.00 in the second. The intensive care mortality rate for COVID-19 patients who develop ventilator-associated pneumonia has been reported as 42.7% [9]. The intensive care mortality rates in our study were 16.36% and 38.36% in the first and second waves of COVID-19, respectively (*p* = 0.047). Again, a significant difference was found between both waves. This could be due to the increased ventilator-associated pneumonia rates and invasive pulmonary aspergillosis rates in the second wave.

In our intensive care unit, we found a significant increase in the incidence of ventilator-associated pneumonia and COVID-19 associated pulmonary aspergillosis in the second wave of COVID-19 compared to the first (*p* < 0.001). Steroid use was an independent risk factor for developing ventilator-associated pneumonia and invasive pulmonary aspergillosis. The rate of steroid use went from 29.09% in the first wave to 91.78% in the second (*p* < 0.001). Cona et al. described an increase in bloodstream infections in COVID-19 patients who received steroids without increasing mortality [29]. Yet, several studies have suggested that steroids may not cause worse outcomes. In the RECOVERY trial, corticosteroids were shown to decrease the length of mechanical ventilation [12]. The CoDEX trial showed a decrease in the length of mechanical ventilation. It displayed no increase in the incidence of ventilator-associated pneumonia [13]. In one study, corticosteroids were not shown to increase the rate of ventilator-associated pneumonia or bacteraemia [30]. Giaccobe et al. reported an elevated rate of bloodstream infections in this cohort. Only time will tell if the benefits of using anti-inflammatory medication outweigh the risks [31].

The rate of invasive pulmonary aspergillosis has been less in critically ill COVID-19 patients than in influenza patients [26]. Our results mirror these findings. The rate of invasive pulmonary aspergillosis in this study was 0%, 9.59%, and 13.33% in the first wave of COVID-19, the second wave of COVID-19 and the influenza group, respectively. In contrast, COVID-19 patients have been shown to have an elevated rate of invasive pulmonary aspergillosis compared to non-COVID-19 patients overall [9]. The community-acquired pneumonia group in our study exhibited a rate of 6.82%.

No patients were treated with IL-6 inhibitors or Janus kinase inhibitors. These immunomodulating agents have been theorized to increase the risk of ventilator-associated pneumonia. Yet, one study described how the early use of IL-6 antagonists could possibly decrease the need to intubate and decrease mortality. More research in this area is required [32]. Janus kinase inhibitors have been reported to reduce mortality if given within the first one to two weeks of infection. Again, further evaluation for this medication is required [11]. These therapies could show promise in the future.

It is thought that COVID-19 patients displayed a higher rate of ventilator-associated pneumonia due to reduced resources and lack of personal protective equipment at the beginning of the pandemic. Many patients received care outside of the ICU. These factors may also have increased the rate of underdiagnosis [33,34,35]. We have seen a significant increase in the rate of ventilator-associated pneumonia in the second wave. This could be related to improved levels of staffing and improved diagnosis.

The bacteria cultured from all groups showed a gram-negative predominance. This is in keeping with current systematic reviews. In contrast, *S. aureus* was grown infrequently, which has been reported at higher rates in other studies [8,9,36,37]. This may be due to local growth in this ICU. The second wave cultured *Klebsiella* at an elevated rate compared to the first wave. The reason for this is not entirely clear. It has been theorized that bacterial growth could be related to the severity of the disease [38].

Diagnosis of invasive pulmonary aspergillosis has been described as challenging in the literature, which may lead to under detection [39]. In addition, one study displayed a reduction in the number of bronchoalveolar lavages performed during the pandemic due to fear of viral transmission [40]. This reduction may lead to a further decrease in detection rates. Currently, it is recommended to use invasive sampling methods, such as bronchoalveolar lavage, instead of non-invasive methods, such as endotracheal tube aspiration [41].

Adjuncts such as galactomannan and B-D-glucan can aid in the diagnosis, but clinical judgement remains paramount. Polymerase chain reaction and lateral flow devices can expedite diagnosis [42,43,44,45]. Galactomannan sampled from bronchoalveolar lavage has been shown to increase the accuracy of diagnosis when used alongside the clinical context [42]. The main emphasis is on accurate early diagnosis [45]. This aids in the early recognition of multi-drug resistant organisms, and appropriate early treatment. Improved outcomes have been reported as a result [45].

The limitations of this study included the following. This study is a single centre study with 276 patients. The fact that the community-acquired pneumonia group and the influenza group have been collected from a broader timeframe may also affect results. Radiological criteria were not used in the diagnosis of invasive pulmonary aspergillosis.

## 5. Conclusions

In summary, the COVID-19 pandemic has placed immense strain on healthcare systems around the globe. This disease has created further challenges for clinicians. Ventilator-associated pneumonia and invasive pulmonary aspergillosis have been reported at elevated rates in critically ill COVID-19 patients when compared to non-COVID-19 patients. Several theories have been put forward to explain why COVID-19 patients are at higher risk.

We performed a retrospective chart review of 276 patients from four groups: COVID-19 Wave 1, COVID-19 Wave 2, influenza, and community-acquired pneumonia. When comparing the second wave of COVID-19 to the first wave, a significantly increased mortality rate, ICU length of stay, and length of mechanical ventilation were shown. Significantly elevated rates of ventilator-associated pneumonia and invasive pulmonary aspergillosis were displayed. A significant increase in steroid use accompanied this. Steroids were shown to be an independent risk factor for the development of ventilator-associated pneumonia and invasive pulmonary aspergillosis across all four groups. The rates of invasive pulmonary aspergillosis in the second wave are less than in the influenza group. This is in keeping with the publication by Rouzé et al.

The above findings leave us with a pessimistic view. Despite an increased understanding of this disease, no clinical trials have shown any promising therapeutic options at present. In order to delve deeper into this topic, further studies are required. It would be interesting to undertake a multicentre study investigating these four groups.

## Figures and Tables

**Table 1:** Clinical Characteristics—co-morbidities, prognostic scores, treatment.

N = 276	COVID-19 Wave 1 (*n* = 55)	COVID-19 Wave 2 (*n* = 73)	Influenza (*n* = 60)	Community-Acquired Pneumonia (*n* = 88)	*p*-Value
Male (*n*, %)	38 (69.09%)	48 (65.75%)	33 (55.00%)	56 (63.64%)	0.381
Female (*n*, %)	17 (30.91%)	25 (34.25%)	27 (45.00%)	32 (36.36%)	0.381
Age (years) (mean, [SD])	60.38 [13.65]	64.33 [12.21]	61.70 [16.58]	62.33 [15.13]	0.476
CCF (*n*, %)	6 (10.90%)	5 (6.85%)	6 (10.00%)	10 (11.36%)	0.791
IHD (*n*, %)	13 (23.63%)	9 (12.32%)	25 (41.67%)	10 (11.36%)	<0.001
HTN (*n*, %)	17 (30.90%)	37 (50.68%)	19 (31.67%)	24 (27.27%)	0.012
DM (*n*, %)	12 (21.82%)	17(23.29%)	11 (18.33%)	8 (9.09%)	0.079
COPD (*n*, %)	6 (10.90%)	15 (20.55%)	22 (36.67%)	30 (34.09%)	0.003
Asthma (*n*, %)	7 (12.73%)	9 (12.32%)	7 (11.67%)	7 (7.95%)	0.757
CKD (*n*, %)	7 (12.73%)	3 (4.11%)	3 (5.00%)	4 (4.55%)	0.160
Cirrhosis (*n*, %)	0 (0%)	2 (2.74%)	1 (1.67%)	2 (2.27%)	0.687
Cancer (*n*, %)	1 (1.82%)	10 (13.70%)	7 (11.67%)	13 (14.77%)	0.092
Immunosuppressed * (*n*, %)	5 (9.09%)	12 (16.44%)	9 (15.00%)	15 (17.05%)	0.558
BMI (kg/m^2^) (mean, [SD])	29.80 [16.28]	29.18 [6.58]	27.08 [9.62]	25.79 [9.55]	0.412
SAPSII (mean, [SD])	49.75 [18.63]	41.63 [17.63]	55.73 [17.26]	48.92 [18.83]	<0.001
SOFA worst throughout admission (mean, [SD])	9.18 [4.32]	8.94 [4.77]	11.13 [4.49]	9.76 [4.15]	0.029
Steroids (*n*, %)	16 (29.09%)	67 (91.78%)	37 (61.67%)	52 (59.09%)	<0.001
Treated with antifungals (*n*, %)	14 (25.45%)	35 (47.95%)	17 (28.33%)	28 (31.80%)	<0.001
CPIS (mean [SD])	2.00 [0]	4.46 [3.15]	1.52 [2.62]	6.67 [4.16]	<0.001

CCF: congestive cardiac failure, IHD: ischaemic heart disease, HTN: hypertension, DM: diabetes mellitus, COPD: chronic obstructive pulmonary disease, CKD: chronic kidney disease, BMI: body mass index, SAPSII: Simplified Acute Physiology Score, SOFA: Sequential Organ Failure Assessment, CPIS: Clinical Pulmonary Infection Score. * Immunosuppression criteria used: solid tumour with chemotherapy in the last 3 months, progressive metastatic disease, haematological malignancies, solid organ transplantation, HIV infection (with or without AIDS), corticosteroids (>3 months at any dosage or ≥1 mg/kg prednisone equivalent per day for >7 days), and/or immunosuppressive drugs.

**Table 2:** Outcomes—length of stay, length of mechanical ventilation, ICU mortality, VAP, and IPA.

N = 276	COVID-19 Wave 1 (*n* = 55)	COVID-19 Wave 2 (*n* = 73)	Influenza (*n* = 60)	Community-Acquired Pneumonia (*n* = 88)	*p*-Value
ICU LOS (median, [IQR])	12.00 [5.00, 26.00]	14.00 [6.00, 32.50]	9.00 [3.25, 20.00]	10.00 [4.00, 18.75]	0.010
MV (median, [IQR])	8.00 [0.00, 17.00]	11.00 [0.50, 25.00]	7.00 [1.00, 14.75]	5.00 [0.25, 14.00]	0.009
ICU Mortality (*n*, %)	9 (16.36%)	28 (38.36%)	20 (33.33%)	23 (26.14%)	0.047
VAP (*n*, %)	3 (5.45%)	20 (27.40%)	10 (16.67%)	3 (3.41%)	<0.001
IPA (*n*, %)	0 (0%)	7 (9.59%)	8 (13.33%)	6 (6.82%)	<0.001

LOS: length of stay, MV: mechanical ventilation, VAP: ventilator-associated pneumonia, IPA: invasive pulmonary aspergillosis.

**Table 3:** Microbiological cultures, galactomannan, and B D glucan results.

N = 276	COVID-19 Wave 1 (*n* = 55)	COVID-19 Wave 2 (*n* = 73)	Influenza (*n* = 60)	Community-Acquired Pneumonia (*n* = 88)	*p*-Value
*Acinetobacter baumannii* (*n*, %)	0 (0%)	0 (0%)	1 (1.67%)	0 (0%)	
*Berkholderia* (*n*, %)	1 (1.81%)	0 (0%)	0 (0%)	0 (0%)	
*Citrobacter* (*n*, %)	0 (0%)	1 (1.37%)	0 (0%)	0 (0%)	
*Enterobacter cloacae* (*n*, %)	0 (0%)	1 (1.37%)	2 (3.33%)	0 (0%)	
*Escherichia coli* (*n*, %)	1 (1.81%)	2 (2.74%)	1 (1.67%)	1 (1.14%)	
*Klebsiella* (*n*, %)	1 (1.81%)	7 (9.59%)	0 (0%)	1 (1.14%)	
*Pseudomonas* (*n*, %)	0 (0%)	2 (2.74%)	0 (0%)	1 (1.14%)	
*Staphylococcus aureus* (*n*, %)	0 (0%)	0 (0%)	1 (1.67%)	1 (1.14%)	
*Stenotrophomonas Maltophilia* (*n*, %)	0 (0%)	2 (2.74%)	2 (3.33%)	0 (0%)	
*Streptococcus* (*n*, %)	0 (0%)	1 (1.37%)	0 (0%)	0 (0%)	
Galactomannan (serum) (mean, [SD])	0.089 [0.047]	0.179 [0.306]	0.240 [0.318]	0.108 [0.210]	0.238
Galactomannan (BAL) (mean, [SD])	0.100 [0.082]	0.897 [1.903]	0.730 [1.708]	0.607 [1.510]	0.829
B D glucan (pg/mL) (mean, [SD])	74.62 [51.68]	85.97 [119.38]	178.60 [202.19]	117.37 [150.07]	0.247

BAL: bronchoalveolar lavage.

## Data Availability

Data is contained within the article or Supplementary Materials.

## Supplementary Materials

The following are available online at https://www.mdpi.com/article/10.3390/antibiotics11050632/s1, Table S1: Multivariate analysis for factors involved in the development of VAP, Table S2: Multivariate analysis for factors involved in the development of invasive pulmonary Aspergillosis.
