# Supplementary material for: Elevated Rates of Ventilator-Associated Pneumonia and COVID-19 Associated Pulmonary Aspergillosis in Critically Ill Patients with SARS-CoV2 Infection in the Second Wave: A Retrospective Chart Review
Source: Antibiotics (Basel). 2022 May 7;11(5):632. doi: 10.3390/antibiotics11050632 (PMC9138004; doi:10.3390/antibiotics11050632)
Supplement: Supplementary file 1 [file antibiotics-11-00632-s001.zip › antibiotics-1698400-supplementary.pdf]

Supplementary Material Table S1. Multivariate analysis for factors involved in the development of ventilator-associated pneumonia.

	p value	OR	CI95
**SAPS2(point)**	0.04	1.03	1.01-1.07
**Gender (male)**	0.44	1.37	0.60-3.11
**Corticosteroids**	0.01	5.24	1.11-24.38
**1st Wave COVID-19**	Reference		
**2nd Wave COVID-19**	0.006	7.82	7.88-34.68
**iMV time(days)**	0.15	0.98	0.95-1.07

SAPS: Simplified Acute Physiology Score. *OR*: Odds Ratio. *CI*. Confidence interval. *iMV*: invasive mechanical ventilation.

Supplementary Material Table S2. Multivariate analysis for factors involved in the development of invasive pulmonary aspergillosis.

	p value	OR	CI95
**SAPS2(point)**	0.05	1.04	0.99-1.08
**Gender (male)**	0.53	1.35	0.52-3.49
**Corticosteroids**	0.006	11.11	1.38-9.27
**1st Wave COVID-19**	Reference		
**2nd Wave COVID-19**	0.21	2.05	0.65-6.50
**iMV time(days)**	0.62	0.99	0.96-1.02

SAPS: Simplified Acute Physiology Score. *OR*: Odds Ratio. *CI*. Confidence interval. *iMV*: invasive mechanical ventilation.
